# Association of Matrix Metalloproteinase 9 (*MMP-9*) Polymorphisms with Asthma Risk: A Meta-Analysis

**DOI:** 10.1155/2019/9260495

**Published:** 2019-02-25

**Authors:** Fenfang Zou, Jianpeng Zhang, Guoan Xiang, Hongbin Jiao, Hongmei Gao

**Affiliations:** Department of Respiratory, The Third Medical Center of Chinese People's Liberation Army General Hospital, Beijing, China

## Abstract

Published data on the association between *MMP-9* polymorphisms (−1562 C > T, rs3918242; Gln279Arg, rs17576 Arg668Gln, rs17577) and asthma susceptibility are inconclusive. To derive a more precise estimation of this association, a meta-analysis was performed. A literature search was conducted in PubMed, Web of Science, EMBASE, Wanfang, and China National Knowledge Infrastructure (CNKI) databases to identify eligible studies. The pooled odds ratios (ORs) and corresponding 95% confidence intervals (CIs) were used to calculate the strength of association. Sensitivity analysis was performed to evaluate the influence of individual studies on the overall effect estimates, and funnel plots and Egger's test were inspected for indication of publication bias. Seven studies with 1592 asthma patients and 1987 controls were finally identified. Overall, we found no significant association between −1562 C > T, rs3918242 polymorphism, and asthma susceptibility in any of the genetic model comparisons. After categorizing studies into different subgroups on the basis of ethnicity and age, there is still no significant association. For the Gln279Arg, rs17576 polymorphism, there seems to be a significant association in the allelic genetic model in regard to the *P* value (OR = 1.11, 95% CI = 1.00–1.22, *I* ^2^ = 0%, *P*_(Z)_=0.044); however, the value of lower 95% CI is 1.0. For the Arg668Gln, rs17577 polymorphism, a high significant association was observed in the dominant model comparison (OR = 1.65, 95% CI = 1.28–2.11, *I* ^2^ = 22.50%, *P*_(Z)_=0), recessive model comparison (OR = 2.40, 95% CI = 1.23–4.72, *I* ^2^ = 0%, *P*_(Z)_=0.011), homozygote genotype comparison (OR = 2.69, 95% CI = 1.36–5.33, *I* ^2^ = 0%, *P*_(Z)_=0.004), and allelic genetic model (OR = 1.59, 95% CI = 1.29–1.97, *I* ^2^ = 36.9%, *P*_(Z)_=0). Sensitivity analysis demonstrated the stability of our results, and publication bias was not evident. The present meta-analysis suggests that *MMP-9* Arg668Gln, rs17577 polymorphism may be the risk factor for asthma susceptibility.

## 1. Introduction

Asthma is a chronic respiratory inflammation disease characterized by airway hyperresponsiveness, reversible airway obstruction, and airway wall remodelling, which is associated with significant thickening of the reticular basement membrane and deposition of the extracellular matrix components [1–3]. In this regard, matrix metalloproteinases (MMP) family, which is a Zn^2+^- and Ca^2+^-dependent endopeptidases, plays a vital role in degradation of extracellular matrix components [4].

*MMP-9*, one of the MMP family proteins, is mainly produced by macrophages and neutrophils but also by epithelial cells, mast cells, fibroblasts, and smooth myocytes, which are key mediators in the airway wall remodeling and metabolism of the extracellular matrix [5–7]. Its increased levels were found in blood, sputum, and bronchoalveolar lavage from patients with asthma exacerbation [8, 9]. The ratio of *MMP-9* to its natural inhibitor (TIMP-1) in bronchoalveolar lavage (BAL) fluid was also higher in children with symptomatic asthma, as compared to that of healthy controls [10]. In addition, deficiency of *MMP-9* in mice leads to enhanced allergen-induced airway inflammation and increases the numbers of eosinophils and levels of cytokines such as interleukin (IL)-4 and IL-13 [11–13]. These accumulated data support the idea that *MMP-9* plays an important role in asthma pathogenesis.

The *MMP-9* gene is located on chromosome 20q11.2–q13.1, a position which has been shown to be associated with bronchial hyperresponsiveness and specific sensitization [14, 15]. Up to now, at least twelve potential clinically relevant SNPs were found in the promoter and coding region, which are important for the *MMP-9* expression and function [16–18]. Therefore, a lot of genetic epidemiology studies have assessed the association of *MMP-9* gene polymorphisms and susceptibility of asthma in different populations [19–26]. Most of them focused on a 9-bp sequence containing the −1562 C > T, rs3918242 polymorphic site in the promoter region, which is an important regulatory element [19–21,23,25]. In addition, in the coding region of *MMP-9* gene, the association of Gln279Arg, rs17576 and Arg668Gln, rs17577 polymorphisms with the susceptibility of asthma was also assessed [20–22,26]. There are few association studies between other polymorphisms of *MMP-9* gene and asthma susceptibility. However, these results were inconclusive and inconsistent. Therefore, we performed a meta-analysis of all eligible studies to obtain more precise estimation of the association of *MMP-9* gene polymorphisms including three SNPs (−1562 C > T, rs3918242; Gln279Arg, rs17576, and Arg668Gln, rs17577) with asthma susceptibility.

## 2. Materials and Methods

### 2.1. Publication Search

Publications were searched using the Pubmed, EMBASE, Web of Science, Chinese National Knowledge Infrastructure (CNKI), and Wanfang databases (the last search was conducted on January 30, 2018). The search strategy utilized in our study was as follows: asthma or asthmatic and matrix metalloproteinase 9 or *MMP-9* or Gelatinase B in combination with polymorphism or mutation or variant. Searching was performed in duplicate by two independent reviewers.

### 2.2. Inclusion and Exclusion Criteria

The inclusion criteria of our study were as follows: (1) Any human studies that estimated the prevalence of matrix metalloproteinase 9 polymorphisms and asthma risk were included, which are published in English or Chinese. (2) The genotype distributions or allele frequency of each study should be available for estimating an odds ratio (OR) with 95% confidence interval (CI). (3) There were sufficient results for extraction of data, that is, number of subjects for each genotype in asthma and control groups. Where eligible papers had insufficient information, we contacted authors by e-mail for additional information. Studies were excluded from our meta-analysis if their authors did not provide us with related data.

### 2.3. Data Extraction

The basic information extracted for each study was as follows: name of first author, publication year, country and ethnicity of case control, age of case, sample size, and genotype frequencies in cases and controls. Data were extracted independently and in duplicate by two reviewers who used a standardized data extraction form. Any disagreement was adjudicated by a third author.

### 2.4. Study Quality Assessment and Meta-Analysis Quality Assessment

Newcastle-Ottawa Scale (NOS) was used to assess the quality of the included studies. Item assessed include selection, comparability of case/controls, exposure/outcome, age and gender. The quality scores ranged from 0 to 9. We divided NOS score into three levels (higher quality, score ≥ 7; moderate quality, 4 ≤ score < 7; low quality, score < 4).

### 2.5. Statistical Analysis

Hardy–Weinberg equilibrium (HWE) was assessed for each study by use of Pearson's chi-square test in control groups, and significance was set at *P* < 0.05. The OR with 95% CI was used to assess the strength of the association between *MMP-9* polymorphism and asthma risk. The pooled OR for *MMP-9* polymorphisms and asthma risk was performed for dominant genetic model (AA + Aa vs. aa), recessive genetic model (AA vs. aa + Aa), homozygote genetic model (AA vs. aa), and allele genetic model (A vs. a), respectively. In the current study, the aa genotype was a wild-type, while the AA genotype was a mutant. The heterogeneity was assessed by using *Q*-test and *I* ^2^ test. A *P* value > 0.10 of *Q*-test and *I* ^2^ < 50% indicates a lack of heterogeneity among the studies, then the fixed-effect model was used. Otherwise, the random effect model was used. Subgroup analysis was performed by ethnicity and case age to further explore ethnicity-specific and age-specific effects. Sensitivity analysis was conducted by sequentially excluding one study at a time to examine the effect of each study on the combined result. The funnel plot and Egger's test was used to assess the potential publication bias. All statistical analyses of this meta-analysis were performed using the STATA 11.0 software (State Corporation, College Station, TX, USA).

## 3. Results

### 3.1. Characteristics of the Studies Included in the Meta-Analysis

**Figure 1:**
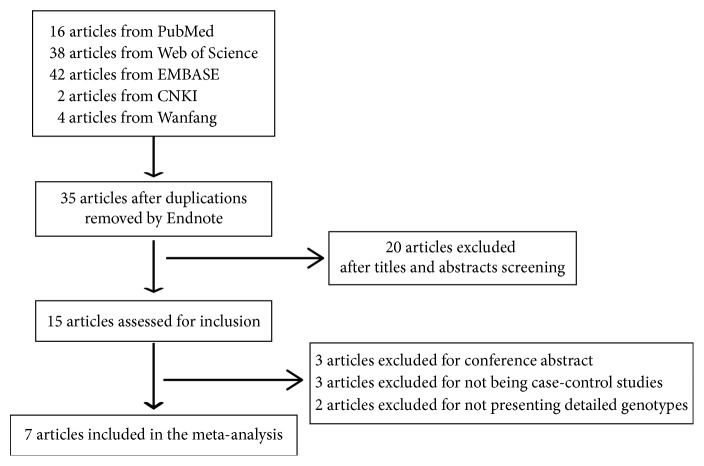
The flow diagram of inclusion of studies in meta-analysis.

The flow diagram in Figure 1 outlined the study selection process. After a comprehensive search of the PubMed, Web of Science, EMBASE, CNKI, and Wanfang databases, a total of 102 articles were identified. Firstly, 67 duplicated studies were excluded by Endnote software, and 35 studies were remained. After reading the abstracts and titles, 20 articles were subsequently excluded because they are review articles or not related the *MMP-9* variants and asthma or not human studies. The remaining 15 articles were then assessed for inclusion. Of these, 8 articles were excluded because 3 articles are conference abstracts, 3 articles lacked a case-control design, and 2 articles lacked detailed genotypes. Eventually, we identified 7 case-control publications, including 1592 asthma patients and 1987 controls, to evaluate the association of *MMP-9* polymorphisms (including three SNPs: −1562 C > T, rs3918242; Gln279Arg, rs17576; and Arg668Gln, rs17577) with asthma susceptibility [19–26]. There are 5 articles on −1562 C > T, rs3918242 polymorphism and two articles on Gln279Arg, rs17576 and Arg668Gln, rs17577 polymorphisms, respectively. For −1562 C > T, rs3918242 polymorphism, three studies were performed in Caucasian and one study was performed in Asian. A study in Turkey did not explain the ethnicity, so we termed it unknown. Two studies were carried out in children and three studies in adults. Polymorphisms in control subjects were in agreement with HWE in all studies (*P* > 0.05). These characteristics of each eligible study are shown in Table 1. The detailed genotype and allele frequencies and HWE examination are listed in Table 2.

### 3.2. Quantitative Synthesis

**Figure 2:**
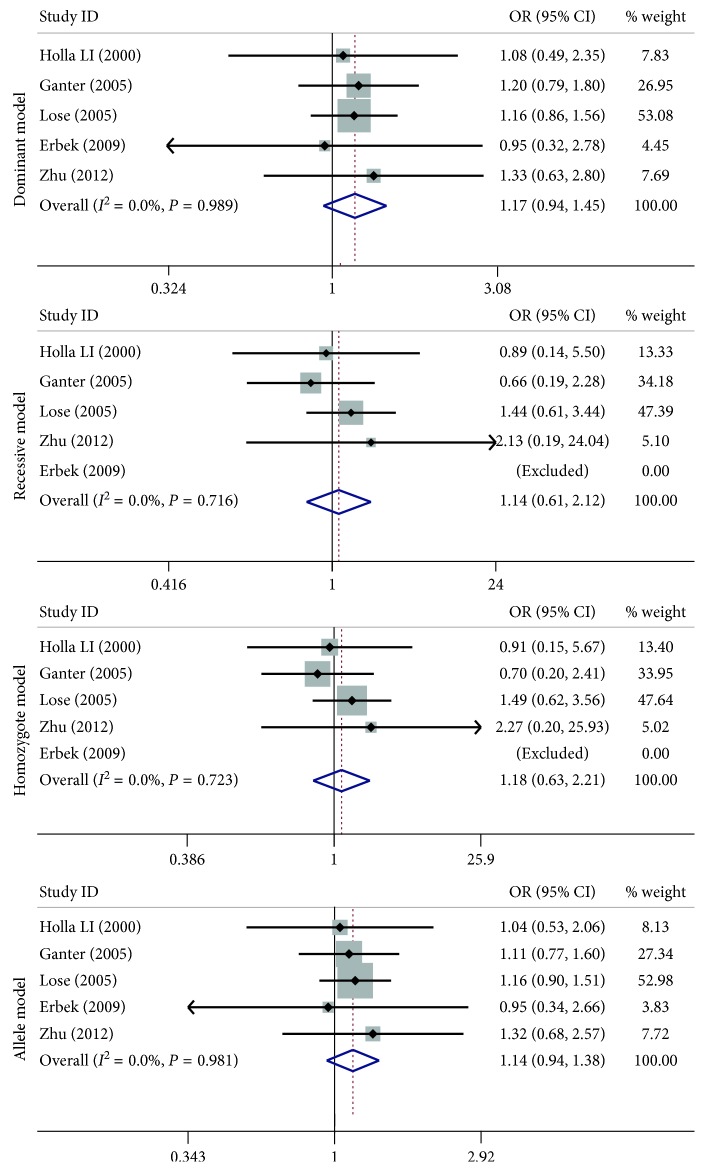
Forest plots of the association between the *MMP-9* −1562 C > T, rs3918242 polymorphism and risk of asthma in the dominant model, recessive model, homozygote model, and allele model comparison.

**Figure 3 fig3:**
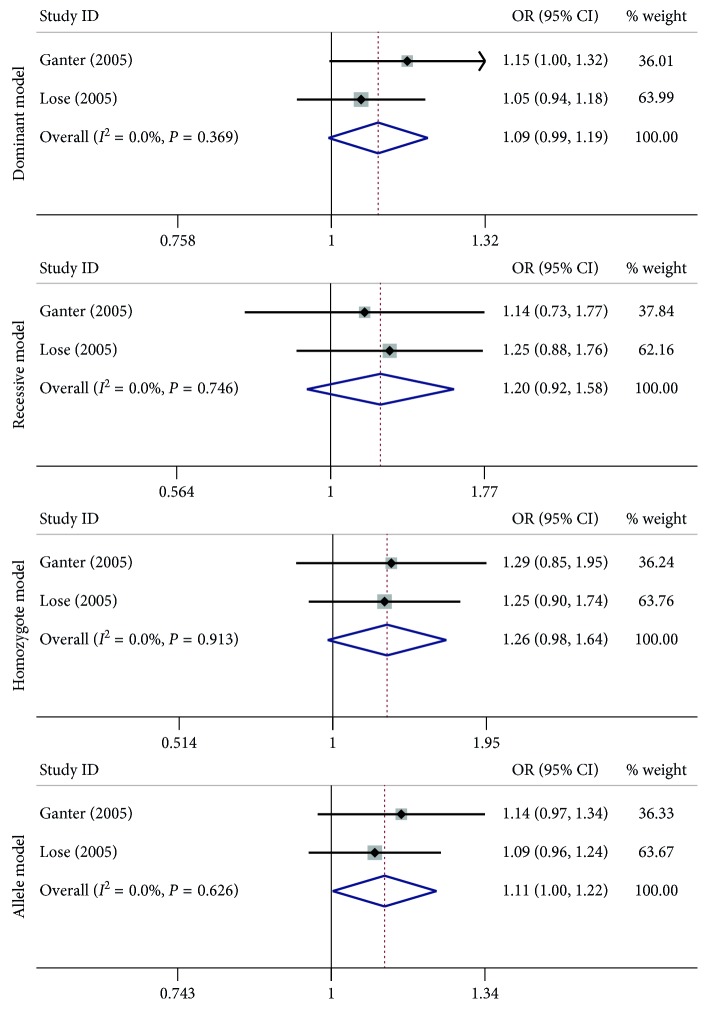
Forest plots of the association between the *MMP-9* Gln279Arg, rs17576 polymorphism and risk of asthma in the dominant model, recessive model, homozygote model, and allele model comparison.

**Figure 4:**
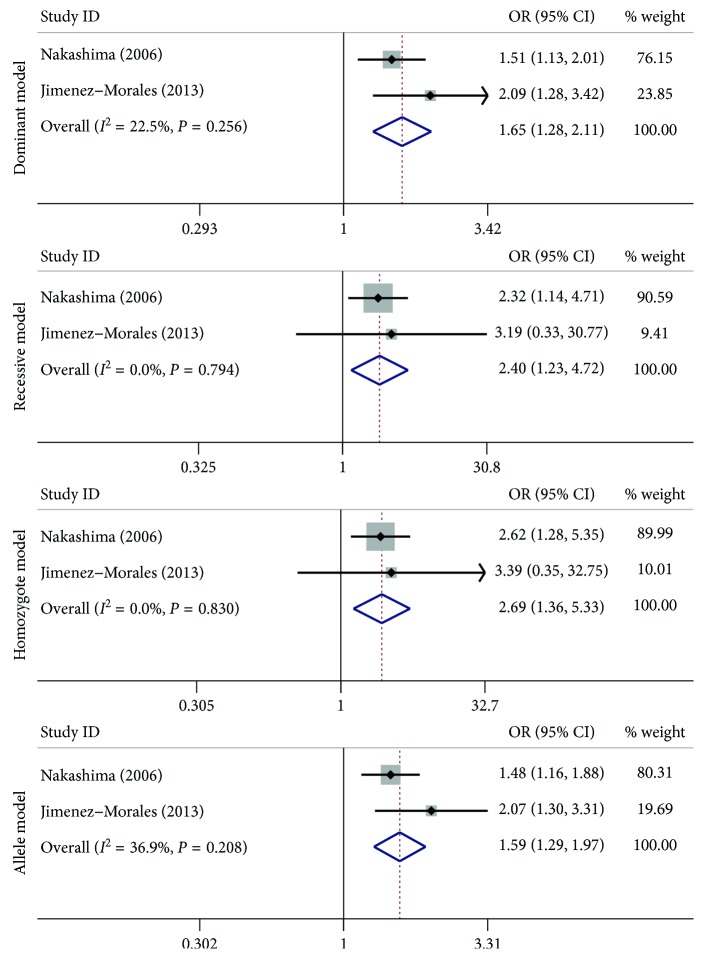
Forest plots of the association between the *MMP-9* Arg668Gln, rs17577 polymorphism and risk of asthma in the dominant model, recessive model, homozygote model, and allele model comparison.

A summary of the meta-analysis findings concerning association between *MMP-9* −1562 C > T, rs3918242; Gln279Arg, rs17576; and Arg668Gln, rs17577 polymorphisms and asthma susceptibility are provided in Figures 2 [Fig fig3]–4 and Table 3. For the −1562 C > T, rs3918242 polymorphism, no significant associations were observed in any of the genetic model comparisons in the overall population. After categorizing studies into different subgroups on the basis of ethnicity and age, the results showed there were still no significant associations between the −1562 C > T, rs3918242 polymorphism and asthma risk (Figure 2 and Table 3). For the Gln279Arg, rs17576 polymorphism, there seemed to be a significant association in the allelic genetic model in regard to the *P* value (OR = 1.11, 95% CI = 1.00–1.22, *I* ^2^ = 0%, *P*_(Z)_=0.044); however, the value of lower 95% CI is 1.0 (Figure 3 and Table 3). For the Arg668Gln, rs17577 polymorphism, there were high significant associations in the dominant model comparison (OR = 1.65, 95% CI = 1.28–2.11, *I* ^2^ = 22.50%, *P*_(Z)_=0), recessive model comparison (OR = 2.40, 95% CI = 1.23–4.72, *I* ^2^ = 0%, *P*_(Z)_=0.011), homozygote genotype comparison (OR = 2.69, 95% CI = 1.36–5.33, *I* ^2^ = 0%, *P*_(Z)_=0.004), and allelic genetic model (OR = 1.59, 95% CI = 1.29–1.97, *I* ^2^ = 36.9%, *P*_(Z)_=0) (Figure 4 and Table 3).

### 3.3. Sensitivity and Publication Bias Analysis

**Figure 5:**
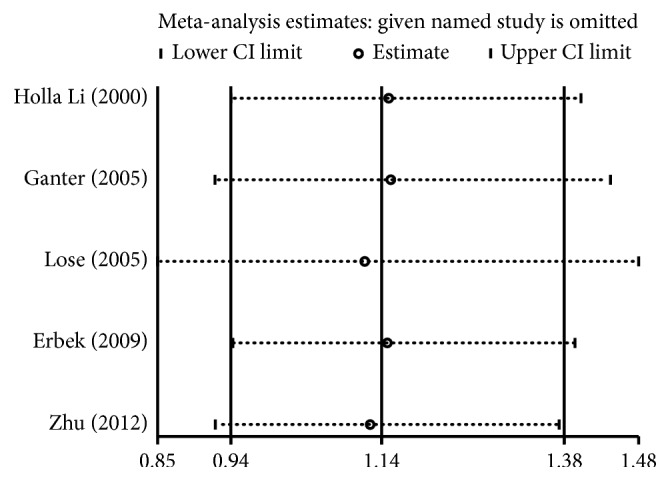
Sensitivity analysis for the *MMP-9* −1562 C > T, rs3918242 polymorphism with asthma susceptibility under the allele model comparison.

**Figure 6:**
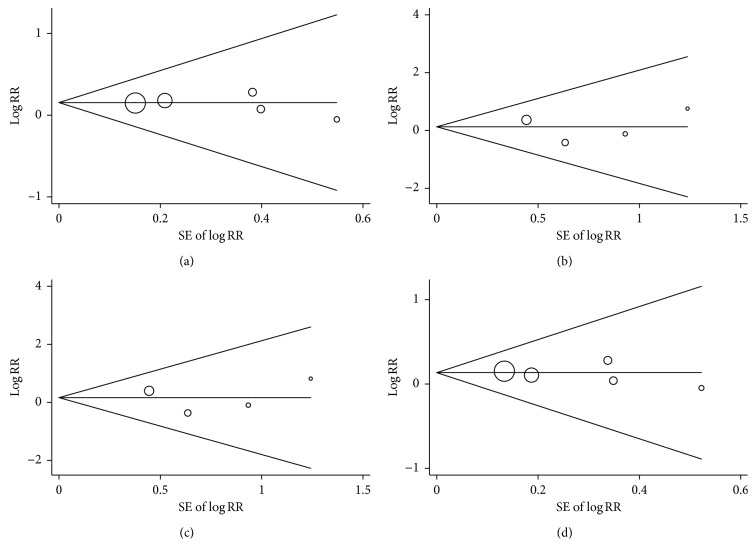
Begg's funnel plot with pseudo 95% confidence limits for publication bias on *MMP-9* −1562 C > T, rs3918242 polymorphism and asthma susceptibility in the dominant model, recessive model, homozygote model, and allele model comparison. (a) Dominant model. (b) Recessive model. (c) Homozygote model. (d) Allele model.

For −1562 C > T, rs3918242 polymorphism, sensitivity analysis was conducted by sequentially excluding individual studies to estimate the stability of the results. After sequentially excluding each study, statistically similar results were found (Figure 5). Potential publication bias was investigated using the funnel plot and was further assessed using Egger's test. The shape of funnel plot (Figure 6) and Egger's test (Supplementary data (available here)) did not indicate any evidence of publication bias for −1562 C > T, rs3918242 polymorphism.

## 4. Discussion

To the best of our knowledge, this is the first meta-analysis to assess the association between *MMP-9* gene polymorphisms and susceptibility of asthma. Our results indicated that there was no significant effect of *MMP-9* −1562 C > T, rs3918242 and Gln279Arg, rs17576 polymorphisms on asthma susceptibility in overall analyses and subgroup analyses. However, for Arg668Gln, rs17577 polymorphism, a significant association with asthma susceptibility was observed in overall analyses.

The −1562 C > T, rs3918242 polymorphism in the promoter sequence is the most frequently studied and best-recognized polymorphism, which is important for gene transcription rate and protein level [16]. However, the current meta-analysis revealed that the −1562 C > T, rs3918242 polymorphism is not in correlation with asthma risk in overall population. After stratified analysis by age or ethnicity, there is still no association with asthma risk. There are some feasible explanations for the lack of the functional association. One is that the elevated *MMP-9* level is not only observed in asthma, but also existed in others inflammatory disease, such as acute respiratory tract diseases and chronic obstructive pulmonary disease (COPD) [27]. So the reason for the elevated MMP-9 level may be caused by other factors, but not by the C > T substitution in the −1562 nucleotide of the MMP-9 promoter. The other reason may be this mutant allele has small effects, but tightly linked to other possibly functional polymorphisms within *MMP-9* or other genes involving in the inflammatory response which play more fundamental roles in asthma. Indeed, one study showed a strong linkage disequilibrium between the −1562 C > T, rs3918242 polymorphism and 2127 *G* > *T*, rs2274755 polymorphism in the fourth intron, increasing the transcriptional level of *MMP-9* and involving in the development of asthma [22]. However, more case-control studies among the different populations are needed to confirm this hypothesis.

Besides the −1562 C > T, rs3918242 polymorphism in the promoter region, we analyzed the other two polymorphisms in the coding region, namely, Gln279Arg, rs17576 and Arg668Gln, rs17577. Gln279Arg, rs17576 and Arg668Gln, rs17577 polymorphisms are single nucleic acid substitutions at positions 836 (A-G) and 2003 (G-A), corresponding to substitutions of glutamine for arginine at amino acid position 279 and arginine for glutamine at amino acid position 668. The Gln279Arg, rs17576 polymorphism is located close to the catalytic region of this enzyme. This change is supposed to modify the affinity of catalytic region of *MMP-9*. The Arg668Gln replacement may result in increased *MMP-9* resistance to its natural inhibitors and higher in vivo activity of this enzyme [28]. In addition, Gln279Arg, rs17576 and Arg668Gln, rs17577 polymorphic variants augment activity of *MMP-9* in children with asthma [29]. These results may suggest a risky role of these two variants in regard to asthma. However, there is a high significant association with asthma susceptibility only for the Arg668Gln, rs17577 polymorphism in this meta-analysis. For Gln279Arg, rs17576 polymorphism, there seems to be a significant association in the allelic genetic model in regard to the *P* value; however, the value of lower 95% CI is 1.0. Therefore, more well-designed and high-quality studies with a larger sample size among the different populations should be conducted to support this finding.

Several limitations should be taken into account when interpreting our results. First, despite a comprehensive search, the number of studies that qualified for inclusion was modest, and the results might be exposed to interference factors such as random error. Second, only published data were included, leading to possible publication bias in this meta-analysis, although no statistically significant publication bias was identified. Third, even though the existing literature had acceptable quality, detailed information was not provided, for e.g., asthma definition varied among different articles, and this may be a confounding factor. In addition, participation rates for cases and controls were not reported in the majority of included studies, thus our meta-analysis was unable to explore the selection bias. Moreover, with limited information about environmental risk factors for asthma, we have no access to evaluate the gene-gene and gene-environmental interactions. Fourth, all the studies emphasize the fact that the differences in polymorphic variation depend on age, ethnicity, and gender, while ignoring the effects of epigenetic modulation.

In conclusion, the current meta-analysis indicates that the *MMP-9* −1562 C > T, rs3918242 and Gln279Arg, rs17576 polymorphisms are not the independent risk factor for asthma susceptibility; however, the Arg668Gln, rs17577 polymorphism is the risk factor for asthma susceptibility.

## Figures and Tables

**Table 1:** Characteristics of the studies included in the meta-analysis.

First author	Year	Country	Ethnicity	Case	Control	Age group	Genotyping method	NOS score
Holla LI	2000	Czech	Caucasian	59	79	Adult	PCR-SSCP	7
Ganter	2005	Germany	Caucasian	231	269	Children	PCR-RFLP	7
Lose	2005	Australian	Caucasian	514	392	Adult	PCR-RFLP	8
Erbek	2009	Turkey	Unknown	30	115	Adult	PCR-RFLP	6
Zhu	2012	China	Asian	65	68	Children	PCR-RFLP	7
Nakashima	2006	Japan	Asian	290	638	Children	Sequenced	8
Jimenez-Morales	2013	Mexico	Hispanic Latino	403	426	Children	TaqMan	9

**Table 2:** Genotype and allele distribution of *MMP-9* polymorphisms in the meta-analysis.

First author	Year	Case			Control			Case		Control		HWE
–1562 C > T		CC	CT	TT	CC	CT	TT	C	T	C	T	*P* value
Holla LI	2000	44	13	2	60	16	3	101	17	136	22	0.168
Ganter	2005	171	56	4	208	54	7	398	64	470	68	0.136
Lose	2005	365	134	15	290	94	8	864	164	674	110	0.906
Erbek	2009	25	5	0	95	20	0	55	5	210	20	0.307
Zhu	2012	44	19	2	50	17	1	107	23	117	19	0.741

Gln279Arg		AA	GA	GG	AA	GA	GG	A	G	A	G	
Ganter	2005	80	117	33	116	119	34	277	183	351	187	0.687
Lose	2005	234	234	75	187	174	45	702	384	548	264	0.637

Arg668Gln		GG	GA	AA	GG	GA	AA	G	A	G	A	
Nakashima,	2006	164	102	16	429	189	16	430	134	1047	221	0.368
Jimenez-Morales	2013	353	47	3	399	26	1	753	53	824	28	0.411

**Table 3:** Results of the pooled and subgroup analyses for the *MMP-9* polymorphism and asthma risk.

SNP	Variable	*n*	Case/control	Dominant model			Recessive model			Homozygote genotype			Allelic		
				OR [95% CI]	*P*_(Z)_	*I* ^2^ (%)	OR [95% CI]	*P*_(Z)_	*I* ^2^ (%)	OR [95% CI]	*P*_(Z)_	*I* ^2^ (%)	OR [95% CI]	*P*_(Z)_	*I* ^2^ (%)
–1562 C > T	Total	5	899/923	1.17 (0.94, 1.45)	0.158	0	1.14 (0.61, 2.12)	0.688	0	1.18 (0.63, 2.21)	0.602	0	1.14 (0.94, 1.38)	0.168	0
	Adult	3	603/586	1.14 (0.87, 1.48)	0.349	0	1.32 (0.61, 2.87)	0.482	0	1.36 (0.62, 2.97)	0.437	0	1.14 (0.90, 1.44)	0.294	0
	Children	3	296/337	1.23 (0.86, 1.76)	0.268	0	0.85 (0.29, 2.48)	0.766	0	0.90 (0.31, 2.63)	0.845	0	1.16 (0.84, 1.60)	0.369	0
	Caucasian	3	804/740	1.16 (0.93, 1.46)	0.193	0	1.08 (0.56, 2.07)	0.809	0	1.12 (0.59, 2.15)	0.725	0	1.14 (0.93, 1.39)	0.219	0

Gln279Arg	Total	2	745/661	1.09 (0.99, 1.91)	0.065	0	1.20 (0.92, 1.58)	0.183	0	1.26 (0.98, 1.64)	0.073	0	1.11 (1.00, 1.22)	**0.044**	0

Arg668Gln	Total	2	693/1064	1.65 (1.28, 2.11)	**0.000**	22.50	2.40 (1.23, 4.72)	**0.011**	0	2.69 (1.36, 5.33)	**0.004**	0	1.59 (1.29, 1.97)	**0.000**	36.90

## Supplementary Materials

Supplementary Materials Publication bias results of Egger's test on *MMP-9* −1562 C > T polymorphism and asthma susceptibility.

## References

[1] Postma D. S., Bleecker E. R., Amelung P. J. (1995). Genetic susceptibility to asthma—bronchial hyperresponsiveness coinherited with a major gene for atopy. *New England Journal of Medicine*.

[2] Westergren-Thorsson G., Larsen K., Nihlberg K. (2010). Pathological airway remodelling in inflammation. *Clinical Respiratory Journal*.

[3] Mauad T., Bel E. H., Sterk P. J. (2007). Asthma therapy and airway remodeling. *Journal of Allergy and Clinical Immunology*.

[4] Crosby L. M., Waters C. M. (2010). Epithelial repair mechanisms in the lung. *American Journal of Physiology-Lung Cellular and Molecular Physiology*.

[5] Kimata M., Ishizaki M., Tanaka H., Nagai H., Inagaki N. (2006). Production of matrix metalloproteinases in human cultured mast cells: involvement of protein kinase C-mitogen activated protein kinase kinase-extracellular signal-regulated kinase pathway. *Allergology International*.

[6] Abel M., Vliagoftis H. (2008). Mast cell-fibroblast interactions induce matrix metalloproteinase-9 release from fibroblasts: role for IgE-mediated mast cell activation. *Journal of Immunology*.

[7] Liang K.-C., Lee C.-W., Lin W.-N. (2007). Interleukin-1*β* induces *MMP-9* expression via p42/p44 MAPK, p38 MAPK, JNK, and nuclear factor-κB signaling pathways in human tracheal smooth muscle cells. *Journal of Cellular Physiology*.

[8] Cataldo D. D., Bettiol J., Noël A., Bartsch P., Foidart J.-M., Louis R. (2002). Matrix metalloproteinase-9, but not tissue inhibitor of matrix metalloproteinase-1, increases in the sputum from allergic asthmatic patients after allergen challenge. *Chest*.

[9] Gagliardo R., La Grutta S., Chanez P. (2009). Non-invasive markers of airway inflammation and remodeling in childhood asthma. *Pediatric Allergy and Immunology*.

[10] Erlewyn-Lajeunesse M. D. S., Hunt L. P., Pohunek P. (2008). Bronchoalveolar lavage *MMP-9* and TIMP-1 in preschool wheezers and their relationship to persistent wheeze. *Pediatric Research*.

[11] McMillan S. J., Kearley J., Campbell J. D. (2004). Matrix metalloproteinase-9 deficiency results in enhanced allergen-induced airway inflammation. *Journal of Immunology*.

[12] Vermaelen K. Y., Cataldo D., Tournoy K. (2014). Matrix metalloproteinase-9-mediated dendritic cell recruitment into the airways is a critical step in a mouse model of asthma. *Journal of Immunology*.

[13] Cataldo D. D., Tournoy K. G., Vermaelen K. (2002). Matrix metalloproteinase-9 deficiency impairs cellular infiltration and bronchial hyperresponsiveness during allergen-induced airway inflammation. *American Journal of Pathology*.

[14] Wjst M., Fischer G., Immervoll T. (1999). A genome-wide search for linkage to Asthma22See the appendix. *Genomics*.

[15] Daniels S. E., Bhattacharrya S., James A. (1996). A genome-wide search for quantitative trait loci underlying asthma. *Nature*.

[16] Zhang B., Ye S., Herrmann S.-M. (1999). Functional polymorphism in the regulatory region of gelatinase B gene in relation to severity of coronary atherosclerosis. *Circulation*.

[17] Ye S. (2006). Influence of matrix metalloproteinase genotype on cardiovascular disease susceptibility and outcome. *Cardiovascular Research*.

[18] Ye S. (2000). Polymorphism in matrix metalloproteinase gene promoters: implication in regulation of gene expression and susceptibility of various diseases. *Matrix Biology*.

[19] Holla L. I., Vasku A., Stejskalova A., Znojil V. (2000). Functional polymorphism in the gelatinase B gene and asthma. *Allergy*.

[20] Ganter K., Deichmann K. A., Heinzmann A. (2005). Association study of polymorphisms within matrix metalloproteinase 9 with bronchial asthma. *International Journal of Immunogenetics*.

[21] Lose F., Thompson P. J., Duffy D., Stewart G. A., Kedda M. A. (2005). A novel tissue inhibitor of metalloproteinase-1 (TIMP-1) polymorphism associated with asthma in Australian women. *Thorax*.

[22] Nakashima K., Hirota T., Obara K. (2006). A functional polymorphism in *MMP-9* is associated with childhood atopic asthma. *Biochemical and Biophysical Research Communications*.

[23] Erbek S. S., Yurtcu E., Erbek S., Sahin F. I. (2009). Matrix metalloproteinase-9 promoter gene polymorphism (−1562 C > T) in nasal polyposis. *American Journal of Rhinology and Allergy*.

[24] Pinto L. A., Depner M., Klopp N. (2010). *MMP-9* gene variants increase the risk for non-atopic asthma in children. *Respiratory Research*.

[25] Hong Z., Lin Y. M., Qin X., Peng J. L. (2011). Serum *MMP-9* is elevated in children with asthma. *Molecular Medicine Reports*.

[26] Jiménez-Morales S., Martínez-Aguilar N., Gamboa-Becerra R. (2013). Polymorphisms in metalloproteinase-9 are associated with the risk for asthma in Mexican pediatric patients. *Human Immunology*.

[27] Brajer B., Batura-Gabryel H., Nowicka A., Kuznar-Kaminska B., Szczepanik A. (2008). Concentration of matrix metalloproteinase-9 in serum of patients with chronic obstructive pulmonary disease and a degree of airway obstruction and disease progression. *Journal of Physiology and Pharmacology*.

[28] Nagase H., Visse R., Murphy G. (2006). Structure and function of matrix metalloproteinases and TIMPs. *Cardiovascular Research*.

[29] Grzela K., Zagórska W., Krejner A. (2016). Polymorphic variants 279R and 668Q augment activity of matrix metalloproteinase-9 in breath condensates of children with asthma. *Archivum Immunologiae et Therapiae Experimentalis*.

